# Incidence of thromboembolic events in asymptomatic carriers of IgA anti ß2 glycoprotein-I antibodies

**DOI:** 10.1371/journal.pone.0178889

**Published:** 2017-07-20

**Authors:** Carlos Tortosa, Oscar Cabrera-Marante, Manuel Serrano, José A. Martínez-Flores, Dolores Pérez, David Lora, Luis Morillas, Estela Paz-Artal, José M. Morales, Daniel Pleguezuelo, Antonio Serrano

**Affiliations:** 1 Department of Immunology Instituto de Investigación, Hospital Universitario 12 de Octubre, Madrid, Spain; 2 Department of Epidemiology, Instituto de Investigación, Hospital Universitario 12 de Octubre, Madrid, Spain; 3 Department of Rheumatology, Instituto de Investigación, Hospital Universitario 12 de Octubre, Madrid, Spain; Keio University, JAPAN

## Abstract

**Background:**

The antiphospholipid syndrome (APS) is defined by simultaneous presence of vascular clinical events and antiphospholipid antibodies (aPL). The aPL considered as diagnostics are lupus anticoagulant and antibodies anticardiolipin (aCL) and anti-ß2 glycoprotein-I (aB2GP1). During recent years, IgA aB2GP1 antibodies have been associated with thrombotic events both in patients positive, and mainly negative for other aPL, however its value as a pro-thrombotic risk-factor in asymptomatic patients has not been well defined.

**Objective:**

To test the role of IgA anti B2GP1 as a risk factor for the development of APS-events (thrombosis or pregnancy morbidity) in asymptomatic population with a 5-year follow-up.

**Methods:**

244 patients isolated positive for anti-beta2-glycoprotein I IgA (Group-1 study) and 221 negative patients (Group-2 control) were studied. All the patients were negative for IgG and IgM aCL.

**Results:**

During the follow-up, 45 patients (9.7%) had APS-events, 38 positive for IgA-aB2GP1 and 7 negative (15.6% vs 3.2%, p<0.001).

The incidence rate of APS-events was 3.1% per year in IgA-aB2GP1 positive patients and 0.6% per year in the control group. Arterial thrombosis were the most frequent APS-events (N = 25, 55%) and were mainly observed in Group-1 patients (21 vs 4, p = 0.001). Multivariate analysis were shown as independent risk-factors for the development of APS-events, age, sex (men) and presence of IgA-aB2GP1 (odds ratio 5.25, 95% CI 2.24 to 12.32).

**Conclusion:**

The presence of IgA-aB2GP1 in people with no history of APS-events is the main independent risk factor for the development of these types of events, mainly arterial thrombosis.

## Introduction

Antiphospholipid syndrome (APS) is a systemic autoimmune disorder defined by the simultaneous presence of antiphospholipid antibodies (aPL) and at least one clinical feature defined in the International consensus diagnostic criteria: vascular thrombosis or pregnancy morbidity.[1] There are three different forms of APS: 1) primary APS, which occur as a primary condition (P-APS), 2) APS associated with other systemic autoimmune diseases (SAD-APS), mainly systemic lupus erythematosus (SLE), and 3) catastrophic APS(C-APS), with simultaneous multiorgan failure and high mortality.[2, 3]

Three aPL are considered as laboratory criteria for APS diagnosis: lupus anticoagulant (LA), anticardiolipin antibodies (aCL) and anti-ß2 glycoprotein-I antibodies (aB2GP1). Only IgM and IgG isotypes of aPL were considered in the consensus established in 2004 in *Sydney*, Australia, during the 11th International Congress of aPL.[4] In recent years, the pathogenic and diagnostic value of aB2GP1 of IgA isotype (IgA-aB2GP1) have been gaining acceptance in the scientific community and it has been shown to be prothrombotic in animal models.[5] IgA-aB2GP1 have also been associated with thrombotic events both in patients with but mainly without other aPLs.[5–8] This evidence implies that the determination of IgA-aB2GP1 is useful in the diagnosis of patients with thrombosis. So from the 13^th^ International Congress on Antiphospholipid Antibodies (2010, Galveston, TX), testing for IgA-aB2GP1in patients negative for IgG and IgM isotypes with APS symptoms was recommended.[9]

Most of the studies to determine the incidence of thromboembolic events in aPL carriers were cross-sectional [10, 11] and were performed in people diagnosed with APS with thrombotic antecedents. Thus these studies evaluated the recurrence of thrombotic events.[12–14] There are very few follow-up cohort-studies to value the incidence of thromboembolic events in asymptomatic aPL-carriers (IgG and IgM isotypes). In these studies the ratio of events per year reached to 3.8%. [15–17]

In the case of IgA-aB2GP1 there are two prospective studies that demonstrated a higher incidence of APS events in carriers of these antibodies, although in both cases in patients with special situations: chronic renal disease treated with hemodialysis, and those who received a kidney transplant.[18, 19]

In the present study to determine the incidence of APS events we followed-up a cohort of patients positive for IgA-aB2GP1 without a history of APS-related simptomatology for five years.

## Material and methods

### Study design

This is a historical cohort follow-up case-control based study.

Primary aim: To determine if the presence of IgA-aB2GP1 in patients without symptoms or antecedents of APS pathology is a risk factor for the occurrence of APS-events in a period of 5 years after identification of aPL.

Secondary aim: To compare the value of this factor in relation to other known vascular risk factors.

Ethical issues: The study was approved by the Hospital “12 de Octubre” Clinical Research Ethical Committee (CREC numbers 13/405 and 12/367).

### Study population

#### Selection criteria

Patients for the study were recruited from those who were referred for an antiphospholipid antibodies study to the Immunology department of the Hospital “12 de Octubre” (Madrid, Spain) in the period 2008 to, 2010. For the selection of patients clinical and laboratory criteria were followed.

#### Laboratory criteria

Study group (Group-1): Patients positive for IgA-aB2GP1 and negative for anticardiolipin (IgG and IgM) antibodies.

Control group (Group-2): Negative the three antibodies: for IgA-aB2GP1and anticardiolipin IgG and IgM. A total of 330 patients were randomly selected for each group among those who met the laboratory criteria to be incorporated into the corresponding group.

#### Clinical inclusion criteria (both groups)

Patients without history of suffering any event considered diagnostic of APS or other clinical features associated with this syndrome but not included in the consensus criteria.[4]

Patients without predisposing factors or preventive treatments for thrombotic events (thrombophlebitis, immobility, treatment with antiplatelet agents or anticoagulants or known deficiencies of coagulation factors).[20]

Patients with clinical monitoring, at least once a year, during the 5 years following the extraction.

#### Clinical exclusion criteria

Patients with a previous history of vascular disease.

Patients treated with anticoagulant or antiplatelet drugs during the follow-up period.

Patients who died in the follow-up without APS-events (15 in Group-1, and 3 in Group-2), because most died outside the hospital and causes of death were not sufficiently well documented or were missing.

#### Selection process

Group-1 (IgA-aB2GP1 positive). Of the 330 patients selected randomly between those who met the laboratory criteria, 244 (73.9%) and also met the clinical requirements.

**Fig 1:**
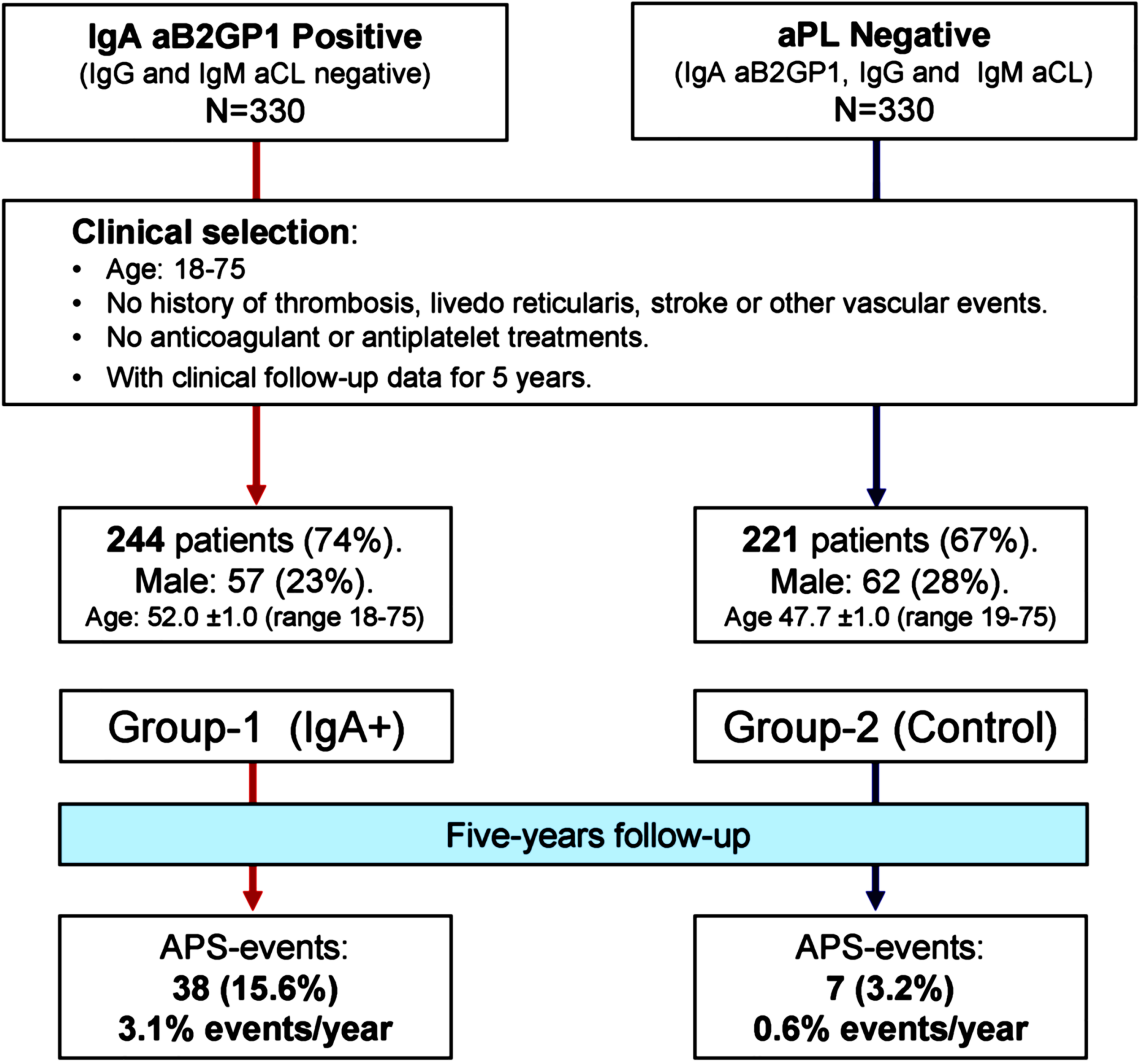
Study disposition and main outcomes.

Group-2 (control). Among the randomly selected 330 patients who met the laboratory conditions, a total of 221 (67.0%) also met the clinical criteria (disposition and main outcomes, Fig 1.).

In relation to ethnicity, more than 95% of the patients in both groups were of European descent.

Clinical Monitoring: The analysis of clinical course to identify thrombotic and cardiovascular events over the next five years was carried out retrospectively.

### Definitions

Thrombotic events: were defined following the International consensus statement on the classification criteria for Antiphospholipid Syndrome^1^ as venous thrombosis, arterial thrombosis and pulmonary thromboembolism, diagnosed clinically and confirmed by images techniques and/or by histopathology study. Arterial thrombosis include ischemic stroke, transient ischemic attack, retinal artery occlusion, myocardial infarction and peripheral arterial occlusion.[21] Pulmonary embolism is considered as venous thromboembolism.[22]

APS-event: any clinical event considered as diagnostic criteria for APS (pregnancy morbidity or thrombosis).[4]

APL-consensus: antiphospholipid antibodies recognized in the Sidney international consensus: lupus anticoagulant, IgG an IgM anticardiolipin and IgG and IgM anti-ß2 glycoprotein-I antibodies.

### Laboratory determinations

The autoantibodies were quantified by enzyme-linked immunosorbent assays (ELISA) using IgG-aCL, IgM-aCL and IgA-aB2GP1 QUANTA Lite (INOVA Diagnostics Inc., San Diego, CA, USA). Antibody levels higher than 20 U/mL were considered positive (99th percentile of a healthy population in our hospital)[6]. This cut off coincides with the manufacturer’s guidelines.

### Validación study of the anticardiolipin determination (IgG IgM) as screening for thepresence of APL consensus

The predictive value of the isolated determination of aCL (IgG and IgM) antibodies as screening to identify patients with any consensus-aPL was evaluated in a population of 5245 individuals. This group was made up of all the patients who were referred by their doctors to our laboratory to perform a study of antiphospholipid antibodies over a period of 3 years.

(1-1-2014 TO 12-31-2016). No patients were excluded. In cases where there were more than one sample per patient, only the first determination was evaluated.

Levels of anti B2GP1 and anti cardiolipin of IgG and IgM isotypes were measured using BioPLex 2200 multiplex immunoassay system APLS IgG and IgM (Bio-Rad, Hercules CA, USA). Antibody levels higher than 18-GPL/mL (aCL IgG), 18 MPL/mL (aCL IgM), and 18 U/mL (aB2GP1, IgG/IgM) were considered positive. The cut off values were established following the International Consensus Guidelines on Anticardiolipin and Anti-B2-Glycoprotein I testing with the 99th percentile of the healthy population in our country.

The manufacturer recommendations for cut off values were 20 GPL/mL, 20 MPL/mL and 20 U/mL.

### Database and statistical methods

Clinical data were collected retrospectively by three DM and incorporated into the database. Data for all the patients (selected and rejected) were revised by three other experienced DM and after the revision process, the database was anonymized.

Characteristics of patients were described for the complete series with mean (± standard error) or absolute frequency and percentage.

Data were stratified, first for Group-1 and Group-2, and then, patients who were APS-events positive and negative. Their distributions were compared with Mann-Whitney test, Chi-square test or Fisher’s exact test samples when appropriate.

Accumulation of events as a function of time was calculated using the Kaplan-Meier method and tested between Group-1 and Group-2 with the Log-rank test.

Logistic regression analysis was used to estimate the association between the risk factors and the outcome (APS-events).[23] Unadjusted and adjusted odds ratios (OR) and 95% CIs are presented for the final set of variables of the predictive model. Model discrimination was quantified using an area under a receiver operating characteristic (ROC) curve. Different cut off points of the predicted probabilities were evaluated in terms of sensitivity, specificity, likelihood ratio positive and negative with their corresponding confidence intervals. Optimal cut off was determined using the maximum value of Youden’s Index.

Data were processed and analyzed using Medcalc for Windows version 16.8.4 (MedCalc Software, Ostend, Belgium).

## Results

### Group characteristics

Clinical characteristics of both groups are shown in Table 1. Age of patients in Group-1 (mean 52.0 ± 1.0, range 18–75 years) was significantly higher (p <0.001) than that observed in Group-2 (47.7 ± 1.0, range 19–75 years). A greater proportion of patients with arterial hypertension (AH) (34.8% vs 22.2%, p = 0.003) and dyslipidemia (23.0% vs 10.9%, p = 0.001) was seen in Group-1.

**Table 1:** Clinical characteristics of patients in both groups. N.S.: Non significant.

	Group-1 N = 244		Group-2 N = 221		
CONDITION	N/Mean	% /SE(range)	N/Mean	% /SE(range)	p
Sex (male)	57	(23.4%)	62	(28.1%)	N.S.
Age (years)	52.0	1.0(18–75)	47.7	1.0(19–75)	0.001
Hypertension	85	(34.8%)	49	(22.2%)	0.003
Diabetes mellitus	29	(11.9%)	16	(7.2%)	N.S.
Type 1 Diabetes	3	(1.2%)	1	(0.5%)	N.S.
Type 2 Diabetes	26	(10.7%)	15	(6.8%)	N.S.
Dyslipidemia	56	(23%)	24	(10.9%)	0.001
Smoker	13	(5.3%)	20	(9%)	N.S.
Ex-smoker	13	(5.3%)	5	(2.3%)	N.S.
Familial antecedents of APS events	4	(1.6%)	1	(0.5%)	N.S.
Aim of the aPL study / underlying disease					
Health screening (without pathology)	26	(10.7%)	44	(19.9%)	0.005
Systemic autoinmune diseases	84	(34.4%)	62	(28.1%)	N.S.
*Systemic lupus erythematosus*	*43*	* (17*.*6%)*	*26*	* (11*.*8%)*	N.S.
*Rheumatoid arthritis*	*17*	* (7%)*	*14*	* (6*.*3%)*	*N*.*S*.
*Mixed conective disease*	*0*	* (0%)*	*2*	* (0*.*9%)*	*N*.*S*.
*Systemic sclerosis*	*14*	* (5*.*7%)*	*13*	* (5*.*9%)*	*N*.*S*.
*Sjögren's syndrome*	*10*	* (4*.*1%)*	*7*	* (3*.*2%)*	*N*.*S*.
Infertility and obstetric studies	11	(4.5%)	11	(5%)	N.S.
Ankylosing spondylitis	2	(0.8%)	2	(0.9%)	N.S.
Muscular diseases	6	(2.5%)	4	(1.8%)	N.S.
Non systemic autoimmunity	10	(4.1%)	8	(3.6%)	N.S.
Neurologic evaluation	7	(2.9%)	12	(5.4%)	N.S.
Endocrinology evaluation	4	(1.6%)	1	(0.5%)	N.S.
Venous insufficiency	6	(2.5%)	8	(3.6%)	N.S.
Other illness	88	(36.1%)	69	(31.2%)	N.S.

The proportion of individuals without any pathology at time of recruitment (the decision to determine the aPL was made in the context of health screening) was significantly higher in Group-2. Other characteristics such as sex, diabetes, and other associated diseases showed no significant differences between groups (Table 1).

**Fig 2:**
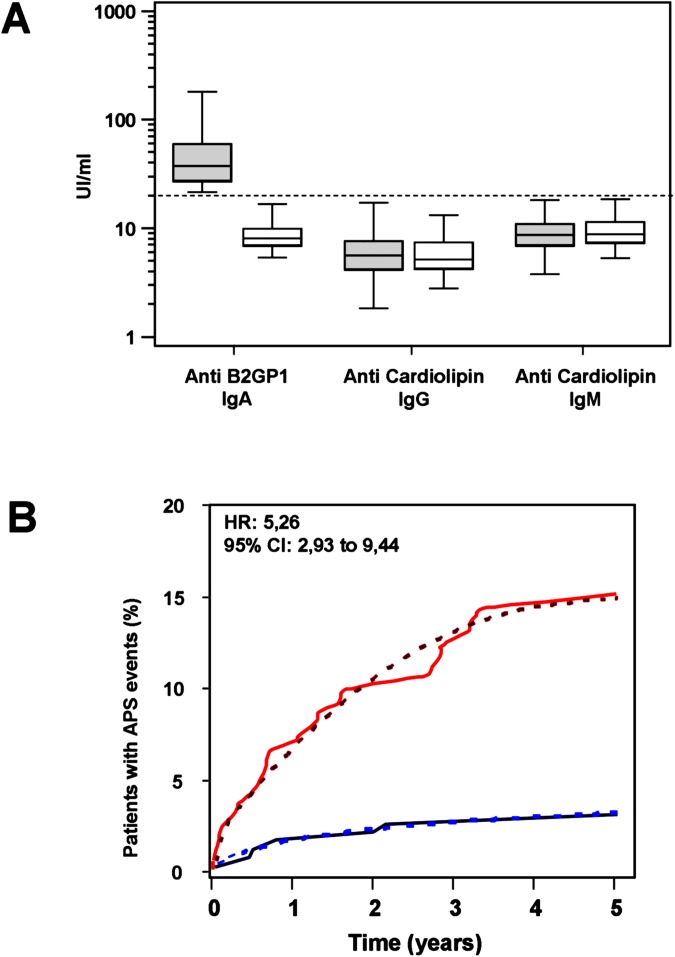
**A. Levels of IgA anti B2GP1 and IgG and IgM anticardiolipin antibodies in Group-1 (grey) and in Group-2 (white). Dotted line corresponds to the cut-off.** B. Accumulation of events as a function of time (p<0.001) in group 1 (red) and Group-2 (blue). Dotted lines represent trends.

No significant differences were found in levels of anti cardiolipin antibodies of IgG and IgM isotypes between patients in Group-1 and Group-2 (Fig 2A).

### Five-year follow-up

During this period, from the total of 465 patients studied in both groups, 45 patients (9.7%) had clinical events considered as clinical criteria for APS diagnosis. These APS-events (Table 2) were significantly more frequent in the Group-1 (15.6%, odds ratio 5.64, p<0.001) than in Group-2 (3.2%). The rate of APS-events per year was also higher in Group-1 (3.1%) compared with Group-2 (0.6%).

**Table 2:** APS events during the five years of the follow-up. N.S.: Non significant.

	Total		Group-1 N = 244		Control N = 221		P	Odds ratio	95% CI
CONDITION	N	%	N	%	N	%			
Total APS events	45	(9.7%)	38	(15.6%)	7	(3.2%)	<0.001	5.64	2.46 to 12.91
Venous thrombosis	18	(3.9%)	15	(6.1%)	3	(1.4%)	0.008	4.76	1.36 to 16.67
*Deep venous thrombosis*	12	(2.6%)	10	(4.1%)	2	(0.9%)	*0*.*028*	*4*.*68*	*1*.*01 to 21*.*60*
*Pulmonary embolism*	6	(1.3%)	5	(2%)	1	(0.5%)	N.S.	*—*	*—*
Arterial thrombosis	25	(5.4%)	21	(8.6%)	4	(1.8%)	0.001	5.11	1.73 to 15.13
*Peripheral arteries*	*4*	* (0*.*9%)*	*3*	* (1*.*2%)*	*1*	* (0*.*5%)*	N.S.	*—*	*—*
*Stroke*	*13*	* (2*.*8%)*	*11*	* (4*.*5%)*	*2*	* (0*.*9%)*	*0*.*022*	*5*.*17*	*1*.*13 to 23*.*59*
*Myocardial infarction*	*8*	* (1*.*7%)*	*7*	* (2*.*9%)*	*1*	* (0*.*5%)*	*0*.*070 (N*.*S)*	*6*.*5*	*0*.*79 to 53*.*24*
Gestational morbidity *	2	(0.4%)	2	(0.8%)	0	(0%)	N.S.	—	—

Consequently the hazard ratio for accumulation of events (Fig 2B) in patients positive for IgA-aB2GP1 was 5.26 (95% CI: 2.93 to 9.44).

### Patients with APS-events

Arterial thrombosis (AT) were the most frequent APS-event (N = 25, 55% of APS events) and were mainly observed in patients in Group-1 (odds ratio 5.11, 95% CI: 1.73 to 15.13).Venous thrombosis were also more frequently observed in patients positive for IgA-aB2GP1 (odds ratio 4.76, 95% CI: 1.36 to 16.67; Table 2).

The 45 patients with APS-events, compared to those who did not present these events (Table 3), were older: 60.4±2.0 years (range 25–75) vs 48.9±0.7 years (range 18–75), p <0.001), male (44.4% vs 23.6%, p = 0.002), and diagnosed with diabetes mellitus, (24.4% vs 8.1%, = 0.001) dyslipidemia (31.1% vs 15.7%, p = 0.009) and hypertension (51.1% vs 26.5%, p <0.001). In addition to these classic risk factors, there was also a higher proportion of patients positive for IgA-aB2GP1 (84.4% vs 49%, p < 0.001).

**Table 3:** Characteristics of the patients who suffer an APS event during the follow up versus patients without APS events. N.S.: Non significant.

	APS events N = 45		Without events N = 420			
CONDITION	N	% /SE(range)	N	% /SE (range)	p	OR
Sex (male)	20	(44.4%)	99	(23.6%)	0.002	2.59
Age (years)	60.4	2.0((25–75)	48.8	0.7(18–75)	<0.001	*—*
Hypertension	23	(51.1%)	111	(26.4%)	<0.001	2.91
Diabetes mellitus	11	(24.4%)	34	(8.1%)	<0.001	3.67
Type 1 Diabetes	*2*	* (4*.*4%)*	*2*	* (0*.*5%)*	*0*.*048*	*—*
Type 2 Diabetes	*9*	* (20%)*	*32*	* (7*.*6%)*	*0*.*005*	*—*
Dyslipidemia	14	(31.1%)	66	(15.7%)	0.009	2.42
IgA-aB2GP1 positive	38	(84.4%)	206	(49%)	<0.001	5.64
Smoker	3	(6.7%)	30	(7.1%)	N.S.	*—*
Ex-smoker	3	(6.7%)	15	(3.6%)	N.S.	*—*
Familial antecedents of APS events	0	(0%)	5	(1%)	N.S.	*—*
Aim of the study / underlying disease						
Health screening (without pathology)	8	(17.8%)	62	(14.8%)	N.S.	*—*
Systemic autoinmune diseases	9	(20%)	137	(32.6%)	N.S.	*—*
*Systemic lupus erythematosus*	*6*	* (13*.*3%)*	*63*	* (15%)*	*N*.*S*.	*—*
*Rheumatoid arthritis*	*1*	* (2*.*2%)*	*30*	* (7*.*1%)*	*N*.*S*.	*—*
*Mixed conective disease*	*0*	* (0%)*	*2*	* (0*.*5%)*	*N*.*S*.	*—*
*Systemic sclerosis*	*2*	* (4*.*4%)*	*25*	* (6%)*	*N*.*S*.	*—*
*Sjögren's syndrome*	*0*	* (0%)*	*17*	* (4%)*	*N*.*S*.	*—*
Infertility and obstetric studies	2	(4.4%)	20	(4.8%)	N.S.	—
Ankylosing spondylitis	0	(0%)	4	(1%)	N.S.	—
Muscular diseases	2	(4.4%)	8	(1.9%)	N.S.	—
Non-systemic autoimmunity	2	(4.4%)	16	(3.8%)	N.S.	—
Neurologic evaluation	1	(2.2%)	18	(4.3%)	N.S.	—
Endocrinology evaluation	0	(0%)	5	(1.2%)	N.S.	—
Venous insufficiency	2	(4.4%)	12	(2.9%)	N.S.	—
Other illness	19	(42.2%)	138	(32.9%)	N.S.	—

No significant differences were observed in patients with systemic autoimmune diseases or any other underlying disease (Table 3).

The female in reproductive age (<45 years) were 147: 70 in Group-1 and 77 in control group. Only two of these 147 women had gestational morbidity: one had an unexplained abortion at 12 weeks of gestation and the other had three abortion of less than 10 weeks during the follow-up. Both women were IgA-aB2GP1 positive.

### Antibodies IgA-aB2GP1 are an independent risk factor for APS-events

Presence of IgA-aB2GP1 and other classical cardiovascular risk factors (age, gender-male, diabetes, dyslipidemia and AH) were identified in univariate analysis as significant mediating factors associated with APS-events (Table 3). Variables which showed a signification p value lower than 0.01 (presence of IgA-aB2GP1, age, gender-male, diabetes and AH) were analyzed in a logistic regression multivariate model of five variables (area under the ROC curve: 0.811; 95% CI: 0,773 to 0,846). Age (OR 1.05, p<0.001), male (OR 2.36, p = 0.020) and IgA-aB2GP1 (OR 5.15, p<0.001) were identified as independent risk factors for APS-events (Table 4A).

**Table 4:** Multivariate análisis to APS-events associated-factors. A. Model with the five most significant variables (selection criteria p<0.01). B. Model replacing the previous model less-significant variable (hypertension) by dyslipidemia. C. Model with the three significant variables in the previous models.

**A.) Multivariate analysis with the five most significant variables**						
**Variable**	**Univariate**			**Multivariate**		
	**Odds ratio**	**95% CI**	**P**	**Odds ratio**	**95% CI**	**P**
IgA-aB2GP1 positive	5.64	2.46 to 12.91	<0.001	5.15	2.19 to 12.14	<0.001
Age (year)	1.06	1.04 to 1.09	<0.001	1.05	1.02 to 1.08	<0.001
Sex (male)	2.59	1.38 to 4.87	0.003	2.36	1.17 to 4.76	0.020
Diabetes mellitus	3.67	1.71 to 7.89	<0.001	1.67	0.69 to 4.07	0.260
Arterial Hypertension	2.91	1.56 to 5.42	<0.001	1.32	0.62 to 2.79	0.480
**B) Alternate 5-variables model including Dyslipidemia (by Arterial Hypertension)**						
**Variable**	**Univariate**			**Multivariate**		
	**Odds ratio**	**95% CI**	**P**	**Odds ratio**	**95% CI**	**P**
IgA-aB2GP1 positive	5.64	2.46 to 12.91	<0.001	5.14	2.18 to 12.15	<0.001
Age (year)	1.06	1.04 to 1.09	<0.001	1.05	1.02 to 1.08	<0.001
Sex (male)	2.59	1.38 to 4.87	0.003	2.28	1.14 to 4.54	0.020
Diabetes mellitus	3.67	1.71 to 7.89	<0.001	1.85	0.79 to 4.36	0.160
Dyslipidemia	2.42	1.22 to 4.8	0.011	1.03	0.48 to 2.21	0.940
**C) Significant variables in all models. (Constant: -6.478)**						
**Variable**	**Coefficient**	**Std. Error**	**Wald**	**Odds ratio**	**95% CI**	**P**
IgA-aB2GP1 positive	1.658	0.435	14.525	5.25	2.24 to 12.32	<0.001
Age (year)	0.051	0.013	14.361	1.05	1.02 to 1.08	<0.001
Sex (male)	0.903	0.346	6.815	2.47	1.25 to 4.86	0.009

In the univariate analysis, dyslipedimia was also identified as a significant mediating factor with a p value of 0.009, so we evaluated a new model of 5 variables in which AH, which was the variate with less signification in the previous model, was replaced by dyslipidemia. The same three variables: age (OR:1.05, p<0.001), gender-male (OR 2.28, p = 0.020) and IgA-aB2GP1 (OR 5.14, p<0.001) remained as independent risk factors for APS-events (Table 4B). The validity of the multivariable analysis with 5 variables from 45 events (9.5 events per variable)[24] was confirmed in 6 models of 4-variables performed with combinations of all significant variables in the univariate analysis. (S1 Table).

In a simpler model with the 3 variables that in all previous models behaved as independent (Table 4C), we were able to obtain an area under the ROC curve of 0.804 (95% CI: 0,765 to 0,838) and the three variables: age (OR:1.05, p<0.001), gender-male (OR 2.47, p = 0.009) and IgA-aB2GP1 (OR 5.25, p<0.001) remained as independent risk factors.

### APS events and systemic autoimmune diseases

The number of patients with SAD was 149. No significant differences were found in the proportion of patients with SAD between Group-1(N = 84, 34.4%) and Group-2 (N = 62, 28,1%, Table 1).

The only differences between patients with SAD (in both groups) compared to patients without SAD were that in those with SAD the proportion of males was significantly lower (16% vs 38.4%, p<0.001) and were older (mean 53.9±1.1 vs 47.9±0.9 years, p<0.001).

Among patients with SAD, the number of APS-events was lower: 9 (6.1%) vs 36(11.3%) than in non-SAD patients, but these differences were not significant (p = 0.083).

### Predictive model for 5-year risk of a first APS-event

Based in the three-variables model described in the multivariable analysis (Table 4-C), an easy risk-calculator for APS-events, where we predicted probabilities of the APS event for a patient, can be established using the formula p = 100*(1/(1+Exp(-6.478+ [1.658*IgA-aB2GP1 positive]+[0.051*age in years]+ [0.903*sex]))).[25] For each patient, in this formula the value of IgA-aB2GP1 positive is 1 (0 for IgA-aB2GP1 negative) and the value of gender-male was 1 (0 for female). For example, a male aged 50 and IgA-aB2GP1 positive has an estimated probability of 20.3% to develop an APS-event.

The ROC analysis of this three-variables predictive-model presents an area under the curve of 0,804 (95% CI:0,765 to 0,839). The Youden’s Index maximum value was 0.50 which corresponded with an optimal cut off[26] of p >10.6.

Using the specificity value, threshold probabilities was established and 3-risk categories were created (S2 Table): moderate (p: > 14.0; specificity <80), high (p: 14.0 to 26.0; specificity 80–95%), and very high (p>26.1, specificity >95%).

Table 5 shows the different values of P according to the characteristics of the patients (age, sex and presence of IgA-AB2GP1), which can be a useful tool to quickly assess the risk for a specific patient.

**Table 5:** Rapid calculation of the risk to undergo a first APS event in the next 5 years of a previously symptomatic patient. The risk is based on the values of the three main risk factors. Moderate risk is marked in orange. high risk in red and very high risk in violet. The values in yellow are close to the cut-off point (10.6).

Age	IgA aB2GP1 POSITIVE		IgA aB2GP1 NEGATIVE	
	Female	Male	Female	Male
20	2.2	5.2	0.4	1
25	2.8	6.6	0.5	1.3
30	3.6	8.4	0.7	1.7
35	4.6	10.6	0.9	2.2
40	5.8	13.3	1.2	2.8
45	7.4	**16.5**	1.5	3.6
50	9.4	**20.3**	1.9	4.6
55	11.8	**24.7**	2.5	5.9
60	**14.7**	**29.8**	3.2	7.5
65	**18.2**	**35.4**	4.1	9.4
70	**22.3**	**41.4**	5.2	11.9
75	**27**	**47.7**	6.6	**14.8**

### Validation of anticardiolipin as screening for aPL consensus

Of the 5245 patients in where the presence of aCL and aB2GP1 of IgG and IgM isotypes IgG was evaluated, 461 (8.8%) were positive for at least one of these aPL and 4783 (91.2%) were negative. The patients aCL positive (IgG or IgM) were 426 (8,1%). Only 35 patients (0.7%) were aCL negative (IgG or IgM) and aB2GPI positive (IgG or IgM).

The negative predictive value (NPV) of an aCL negative value to exclude the presence of any consensus aPL was 99.27% (95% CI: 99.00% to 99.47%). Using the cut off values recommended by the manufacturer of the aPL detection system, the NPV is practically identical (99.26%, 95% CI: 98.99% to 99.46%) and the values of sensitivity, specificity, negative likelihood Ratio and aPL-positive prevalence have minimal variations with respect to the laboratory cut off (Table 6).

**Table 6:** Study for validate aCL antibodies (IgG and IgM) as screening to identify patients positive for aPL consensus (aCL and aB2GP1 IgG/IgM).

Patients studied	Number / %		Women	Men	(%)	p-value
All patients	5245		3411	1834	(35.0%)	-
Positive for consensus aPL with laboratory's cutoff (anti cardiolipin and anti B2GP1 IgG/IgM)						
Anti Cardiolipìn positive (IgG or IgM)	426	(8,1%)	299	127	(29,8%)	0,020
Any consensus aPL positive (IgG or IgM)	461	(8,8%)	324	137	(29,7%)	0,013
Any consensus aPL negative (IgG or IgM)	4784	(91,2%)	3087	1697	(35.5%)	N.S
Anti B2GPI positive (IgG or IgM) and aCL negative (IgG or IgM)	35	(0,7%)	25	10	(28,6%)	N.S:
Value of aCL determination (IgG+IgM) to identify patients with consensus aPL (aCL/aB2GP1 IgG/IgM)						
**Statistic**	Laboratory's cutoff			Manufacturer's cutoff		
	**Value**	**95% CI**		**Value**	**95% CI**	
Sensitivity	92.41%	89.60% to 94.66%		91.80%	88.83% to 94.19%	
Specificity	100%	99.92% to 100%		100%	99.92% to 100%	
Negative Likelihood Ratio	0.08	0.06 to 0.10		0.08	0.06 to 0.11	
Consensus-aPL positive prevalence	8.79%	8.04% to 9.59%		8.37%	7.63% to 9.15%	
Positive Predictive Value	100%	-		100%	-	
Negative Predictive Value	99.27%	99.00% to 99.47%		99.26%	98.99% to 99.46%	

## Discussion

We describe, for the first time, that people with isolated positivity of IgA-aB2GP1 and without previous history of APS-related clinical characteristics (asymptomatic carriers) have a high risk of suffering APS-events independently of other well-know cardiovascular risk factors. The incidence of APS-events in the control group (0.62% per year) was practically the same as was described in the healthy Caucasian population (0.65% per year).[16] Moreover, the incidence of APS-events in the previously asymptomatic IgA-aB2GP1 carriers was 3.1% per year, which was similar to 3.18% per year described for untreated asymptomatic aPL-consensus carriers without any prophylactic treatment[27] and is also within the reported range (1.36–3.8%) described in previous studies assessing the risk of events in asymptomatic aPL-consensus carriers. [28–30] Therefore, isolated positivity of IgA-aB2GPI in asymptomatic patients who are negative for other aPL seems to be an independent risk factor to develop APS-events. This finding is a novelty and may have important clinical implications.

The importance of determining the IgA isotype of anti B2GP1 in patients with clinical signs of APS was established late last century.[31, 32] However until now, there is not the same unanimity to establish the prevalence and clinical significance of IgA-aB2GP1 that which exists for the IgG isotype.[33] Three reasons seem to be responsible for this disagreement: 1) the lack of standardization in diagnostic systems, which results in inhomogeneous outcomes, 2) the need for studies to determinate their utility in the diagnosis and management of patients with APS symptomatology and negative for other aPL, and 3) the lack of prospective studies to evaluate the significance of these antibodies in the asymptomatic population. [34–36]

The first two problems are solved because most of the studies published in recent years use contrasting systems and confirm the usefulness of this isotype in the diagnosis of patients with clinical APS.[37, 38] Our work also contributes to solving the third problem: defining the significance of isolated positivity of IgA with aB2GPI in asymptomatic population.

An interesting finding was that the presence of IgA anti-B2GPI was closely associated with arterial thrombosis than with venous thrombosis. Our work supports the feature previously reported in cross-sectional studies that IgA-aB2GPI is associated with arterial thrombosis both in patients with SLE and without any SAD.[5, 6]

Patients presenting clinical signs of APS which are persistently negative for the consensus-aPL are often labeled as seronegative-APS patients (SN-APS) [29] a concept that, while it is not fully accepted, [39] could serve to label patients that, in our two groups, develop pathology consistent with APS during the follow-up.

Worth noting is that males have a higher risk to develop APS-events than females. This disagrees with other previously published articles where the presence of APS-events in patients with aPL of IgA isotype was more frequent in women. [40] However in most of the series where morbidity in APS and SN-APS was studied, males were clearly under-represented (usually below 20%) and sometimes the sample sizes were insufficient to obtain statistically significant conclusions. [28, 31, 32] Our investigation including 119 men, a substantial sample, could explain this discrepancy. Significantly, in our work, the great majority of patients with APS-events did not suffer from SAD (80%), which could be considered as primary-SN-APS. This finding is similar to that found in studies in our area (Caucasian population), where the patients with APS-events and IgA-aB2GP1 were predominantly without SAD and were mainly male[6] or both sexes were equally represented. [18, 41]

When an asymptomatic patient is positive for aPL consensus, it is possible to establish a pattern of action because there are studies that fixed the risk of aPL positivity and protocols are recommended.[42, 43] However in the presence of isolated IgA-aB2GP1in asymptomatic patients there is no information about the risks. In this respect, our work aims to answer a clinical question that often appears in real medicine: What is the risk of having an APS event in a patient isolate positive for IgA- aB2GP1and without a history of P-APS?

So, we are describing a model to calculate the risk of thrombotic events in isolate positive IgA B2GPI patients according to age, gender and positive IgAB2GPI. This easy risk-calculator could be useful in the outpatient office to establish risk and inform patients. In this model the risk increases with age, especially in males who are positive for IgAaB2GPI. Therefore, this risk may indicate which patients have a higher risk and perhaps could receive prophylactic anticoagulation.

This work has several limitations. Firstly, it is a monocenter study including a population of patients previously studied in our hospital for non-thrombotic pathologies. The ideal study should include healthy people, such as blood donors. Recruiting a large population of 200 healthy individuals isolated positive for IgA-aB2GP1 would be required to make a screening of a large population: as the prevalence of IgA-aB2GP1 in the general population is close to 1%. Evaluation for aPL in around 20,000 individuals would be needed, something that requires resources outside the capacity of a single center. The selected population therefore, may actually represent a clinical advantage. These patients have been tested because their doctors wanted to rule out the possible presence of aPL in the context of a clinical study or a health examination.

Secondly, another limitation is that a complete study with all aPL included in the Sydney consensus was not performed. Diagnostic protocols at Hospital 12 de Octubre consider LA determination only in patients who had a previous thrombotic or obstetric APS-event, and our patients were APS-asymptomatic. Likewise, protocols in force in 2008 for aPL determination only included screening aCL (IgG and IgM). Systematic determination of IgG and IgM anti B2GP1 was introduced in 2013 (LA is still determined only in patients who have had APS events).

The aCL test is more sensitive, but less specific, than aB2GPI, and patients with APS aCL are closely correlated with aB2GPl.[44] Pierangeli and Harris showed that a screening with aCL (IgG and IgM isotypes) and LA test should capture the majority of APS patients.[45] In this context, the validity of the isolated aCL determination in patients in our environment as screening to identify aPL-negative patients has been demonstrated with the study of 5245 patients (APS-symptomatic and asymptomatic). Of the patients who would be considered as aPL negative using the aCL-screening only 0.7% were positive for aB2GP1 (IgG / IgM), a proportion similar to that observed in the general population (1%).[6]

Transferring these data to the two groups in our study, one would expect that this value would be even higher, since both groups are constituted by asymptomatic individuals.

Therefore we can assume that the consensus criteria for diagnosis of APS, both clinical and laboratory, in our two groups are absolutely compatible with those observed in the general asymptomatic population.

Thirdly, a systematical reassessment of aPL levels within the five years of the study was not determined. Most patients were not reevaluated because they were negative for aPL-consensus and the few reassessments were made by decision of the doctors who followed–up on the patients.

In spite of this, we consider our investigation as a novelty because we have been able to demonstrate that the presence of isolate IgA-aB2GPI in aymptomatic patients seems to be a risk factor to develop an APS-event. Also, these results agree with other recent studies that found that the association of IgA-aB2GP1 with the presence of APS-events is practically identical to that observed for the IgG isotype. [46, 47]

So, the inclusion in aPL screening of IgA-aB2GPI may further identify a group of patients with APS-associated clinical manifestations (mostly male), and also with the same risk as IgG-isotype aPL, otherwise missed and the antibodies IgA-aB2GPI may be considered as a laboratory criterion for the diagnosis of APS.

Efforts to standardize IgA-aB2GPI assays are definitely needed. [34, 48–50] Further multicenter cohort study studies with patients of various ethnicities are required, to definitively determine the association between the IgA-aB2GP1 and APS-events in asymptomatic population before recommending preventive treatment with anticoagulants or platelet antiagregants drugs. The proposed formula to calculate patient-risk could be a helpful tool to make clinical decisions, however, before generalizing its use, these rules should be confirmed with multicenter studies and improved with the incorporation of additional markers such as the presence of circulating B2GP1 immune complexes. [51]

## Supporting information

S1 Table Logistic-regression multivariate-analysis using 4-variables models. We performed six models with 4-variables where the two most significant variables described in Table 4 are maintained (IgA-aB2GP1 positive and age). The remaining two variables for each of the six models are combinations of the other four variables that were also demonstrated to be significantly associated with APS-events in the univariate analysis. The three variables defined as independent (IgA-aB2GP1 positive, age and sex) were analyzed in a three-variable model.
(DOC)

S2 Table Limit values of the threshold probability (TP) from which a level of risk is rated. The cut-off value was calculated from Youden’s Index. Patients with p values close to the cutoff and below 14.0 are considered to be in a grey area that could be classified as having an undefined risk. Symbols:+LR and -LR): positive and negative likelihood ratio.
(DOC)

## Abbreviations

CI: *confidence interval*
B2GP1: ß2 glycoprotein-I
aB2GP1: antibodies anti B2GP1
APS: Antiphospholipid syndrome
aPL: Antiphospholipid antibodies
LA: Lupus anticoagulant
aCL: Anticardiolipin antibodies
aB2GP1: Antibodies anti B2GP1
IgA-aB2GP1: Antibodies anti B2GP1 of IgA isotype
HR: Hazard ratio
OR: Odds ratio
SAD: Systemic autoimmune diseases
ROC: Receiver operating characteristic
AH: Arterial hypertension
