# Supplementary material for: Incidence of thromboembolic events in asymptomatic carriers of IgA anti ß2 glycoprotein-I antibodies
Source: PLoS One. 2017 Jul 20;12(7):e0178889. doi: 10.1371/journal.pone.0178889 (PMC5519006; doi:10.1371/journal.pone.0178889)
Supplement: S1 Table — We performed six models with 4-variables where the two most significant variables described in Table 4 are maintained (IgA-aB2GP1 positive and age). The remaining two variables for each of the six models are combinations of the other four variables that were also demonstrated to be significantly associated with APS-events in the univariate analysis. The three variables defined as independent (IgA-aB2GP1 positive, age and sex) were analyzed in a three-variable model. (DOC) [file pone.0178889.s001.doc]

**S1 Table.**

| **Univariate** | **Variable** | **Odds ratio** | **95% CI** | **P** |
| --- | --- | --- | --- | --- |
|  | IgA-aB2GP1 positive | 5.64 | 2.46 to 12.91 | <0.001 |
|  | Age (year) | 1.06 | 1.04 to 1.09 | <0.001 |
|  | Sex (male) | 2.59 | 1.38 to 4.87 | 0.003 |
|  | Diabetes mellitus | 3.67 | 1.71 to 7.89 | <0.001 |
|  | Hypertension | 2.91 | 1.56 to 5.43 | <0.001 |
|  | Dyslipidemia | 2.42 | 1.22 to 4.80 | 0.011 |
| **Multivariate** | **Variable** | **Odds ratio** | **95% CI** | **P** |
| Four variables. Model 1 | **IgA-aB2GP1 positive** | 5.16 | 2.19 to 12.14 | <0.001 |
|  | **Age (year)** | 1.05 | 1.02 to 1.08 | <0.001 |
|  | **Sex (male)** | 2.27 | 1.14 to 4.54 | 0.020 |
|  | Diabetes mellitus | 1.86 | 0.8 to 4.32 | 0.148 |
| Four variables. Model 2 | **IgA-aB2GP1 positive** | 5.2 | 2.21 to 12.23 | <0.001 |
|  | **Age (year)** | 1.05 | 1.02 to 1.08 | 0.002 |
|  | **Sex (male)** | 2.56 | 1.29 to 5.07 | 0.007 |
|  | Hypertension | 1.50 | 0.74 to 3.05 | 0.265 |
| Four variables. Model 3 | **IgA-aB2GP1 positive** | 5.16 | 2.19 to 12.19 | <0.001 |
|  | **Age (year)** | 1.05 | 1.02 to 1.08 | <0.001 |
|  | **Sex (male)** | 2.46 | 1.25 to 4.85 | 0.009 |
|  | Dyslipidemia | 1.13 | 0.53 to 2.4 | 0.749 |
| Four variables. Model 4 | **IgA-aB2GP1 positive** | 4.73 | 2.02 to 11.05 | <0.001 |
|  | **Age (year)** | 1.05 | 1.02 to 1.08 | <0.001 |
|  | Diabetes mellitus | 2.19 | 0.95 to 5.02 | 0.064 |
|  | Dyslipidemia | 1.02 | 0.48 to 2.17 | 0.962 |
| Four variables. Model 5 | **IgA-aB2GP1 positive** | 4.71 | 2.02 to 10.98 | <0.001 |
|  | **Age (year)** | 1.05 | 1.02 to 1.08 | <0.001 |
|  | Diabetes mellitus | 2.08 | 0.88 to 4.92 | 0.094 |
|  | Hypertension | 1.16 | 0.55 to 2.42 | 0.701 |
| Four variables. Model 6 | **IgA-aB2GP1 positive** | 4.69 | 2.01 to 10.94 | <0.001 |
|  | **Age (year)** | 1.05 | 1.02 to 1.08 | <0.001 |
|  | Dislipidemia | 1.04 | 0.48 to 2.26 | 0.911 |
|  | Hypertension | 1.36 | 0.66 to 2.8 | 0.404 |
