# Supplementary material for: Incidence of thromboembolic events in asymptomatic carriers of IgA anti ß2 glycoprotein-I antibodies
Source: PLoS One. 2017 Jul 20;12(7):e0178889. doi: 10.1371/journal.pone.0178889 (PMC5519006; doi:10.1371/journal.pone.0178889)
Supplement: S2 Table — The cut-off value was calculated from Youden’s Index. Patients with p values close to the cutoff and below 14.0 are considered to be in a grey area that could be classified as having an undefined risk. Symbols:+LR and -LR): positive and negative likelihood ratio. (DOC) [file pone.0178889.s002.doc]

**S2 Table.**

| **TP (%)** | **Risk** | **Sensitivity** | **95% CI** | **Specificity** | **95% CI** | **+LR** | **95% CI** | **-LR** | **95% CI** |
| --- | --- | --- | --- | --- | --- | --- | --- | --- | --- |
| 10.8 | Cut-off | 75.6 | 60.5 - 87.1 | 74.1 | 69.6 - 78.2 | 2.91 | 2.3 - 3.7 | 0.33 | 0.2 - 0.6 |
| 14.0 | Moderate | 64.4 | 48.8 - 78.1 | 80.2 | 76.1 - 83.9 | 3.26 | 2.4 - 4.4 | 0.44 | 0.3 - 0.7 |
| 22.0 | High | 46.7 | 31.7 - 62.1 | 89.8 | 86.5 - 92.5 | 4.56 | 3.0 - 6.9 | 0.59 | 0.5 - 0.8 |
| 26.7 | Very High | 26.7 | 14.6 - 41.9 | 95.0 | 92.5 - 96.9 | 5.33 | 2.7 - 9.6 | 0.77 | 0.6 - 0.9 |
